# High TXNIP expression accelerates the migration and invasion of the GDM placenta trophoblast

**DOI:** 10.1186/s12884-023-05524-6

**Published:** 2023-04-10

**Authors:** Rina Sa, Jing Ma, Jie Yang, Dong Fang Li, Jie Du, Jian Chao Jia, Zhi Ying Li, Na Huang, Lamusi A, Rula Sha, Gal Nai, Bayar Hexig, Ji Qing Meng, Lan Yu

**Affiliations:** 1 Department of Clinical Medical Research Center, Inner Mongolia People’s Hospital, Hohhot, 010010 China; 2 Department of Clinical Lab, Mongolia Maternity And Child Health Care Hospital, Hohhot, 010000 China; 3 Department of Gynecology and Obstetrics, Inner Mongolia People’s Hospital, Hohhot, 010010 China; 4 Department of Ophthalmology, Inner Mongolia International Mongolian Hospital, Hohhot, 010000 China; 5 Department of Pharmacology, Inner Mongolia People’s Hospital, Hohhot, 010000 China; 6 Department of Genetics 、 Development and Cell Biology, School of Life Sciences, Inner Mongolia University, Hohhot, 010000 China; 7 The State Key Laboratory of Reproductive Regulation and Breeding of Grassland Livestock, School of Life Sciences, Inner Mongolia University, Hohhot, 010000 China; 8 Department of Endocrine and Metabolic Diseases, Inner Mongolia People’s Hospital, Hohhot, 010010 China

**Keywords:** GDM, Placenta, TXNIP, Migration, Invasion

## Abstract

**Introduction:**

Our previous study has proofed the glucose sensitive gene-thioredoxin-interacting protein (TXNIP) expression was up in the placenta of the patients with gestational diabetes mellitus (GDM), but the pathological mechanisms underlying abnormal TXNIP expression in the placenta of patients with GDM is completely unclear and additional investigations are required to explain the findings we have observed. In the present study, we simulated the high TXNIP expression via introducing the Tet-On “switch” in vitro, approximate to its expression level in the real world, to explore the following consequence of the abnormal TXNIP.

**Methods:**

The expression and localization of TXNIP in the placenta of GDM patients and the health control was investigated via immunofluorescent staining, western blot and RT-qPCR. Overexpression of TXNIP was achieved through transfecting Tet-on system to the human trophoblastic cell line-HTR-8/Svneo cell. TXNIP knockout was obtained via CRISPR-Cas9 method. The cell phenotype was observed via IncuCyte Imaging System and flow cytometry. The mechanism was explored via western blot and RT-qPCR.

**Results:**

The expression level of TXNIP in the GDM placenta was nearly 2–3 times higher than that in the control. The TXNIP located at trophoblastic cells of the placenta. When the expression of TXNIP was upregulated, the migration and invasion of the cells accelerated, but cell apoptosis and proliferation did not changed compared with the control group. Furthermore, the size of the TetTXNIP cells became larger, and the expression level of Vimentin and p-STAT3 increased in the TetTXNIP cells. All the changes mentioned above were opposite in the TXNIP-KO cells.

**Conclusions:**

Abnormal expression of TXNIP might be related to the impairment of the GDM placental function, affecting the migration and invasion of the placental trophoblast cells through STAT3 and Vimentin related pathway; thus, TXNIP might be the potential therapeutic target for repairing the placental dysfunction deficient in GDM patients.

**Supplementary Information:**

The online version contains supplementary material available at 10.1186/s12884-023-05524-6.

## Introduction

One of the most frequent complications of pregnancy is gestational diabetes mellitus (GDM) [1, 2]. The incidence of the abnormal glucose metabolism in pregnant women who will deliver live babies is as much as 16.2%, 86.4% of which will be GDM [3]. Moreover, the risk of preeclampsia is 18% higher in the patients with GDM than that of the healthy pregnant women. The offspring of the GDM patients are more susceptible to such complications as neonatal hypoglycemia, macrosomia, respiratory distress syndrome; and to obesity and insulin resistance in their adulthood [2, 4–6]. Thus, GDM do harm to the health of both generations (mother and child). The placenta of GDM patients undergoes pathological structural changes [7], leading to placental dysfunction and embryonic development disorders. Thus, to understand abnormal placental structure and the subsequent function change of GDM from the perspective of molecular biology is of great importance for improving the clinical treating in the future.

TXNIP locates at 1q21.1 of the human chromosome; and its total length is about 4174 bp, encoding the protein composed of 391 amino acid residues. It is a member of the α-arrestin protein family. TXNIP can be induced by glucose and oxidative stress, and also possess the tumor-suppressing effects [8]. An increasing number of studies have confirmed that TXNIP prevents excessive glucose uptake by binding to glucose transporter 1 (Glut1) and inhibiting the peripheral glucose uptake. Thus, TXNIP might play a regulatory role in maintaining intracellular glucose homeostasis [9, 10]. Overexpression of TXNIP aggravates the excess of glucose, leads to the imbalance of oxidative stress and mitochondrial stress, induces the apoptosis of the islet beta cell and T2DM [11]. Our previous study has verified that the expression of TXNIP increased significantly in the placenta of the patients with GDM as well as in HTR-8 cell line treated with high glucose. The increased expression of TXNIP is closely related to the placental dysfunction in GDM patients [12].

Compared with those in the previous study, more accurate measurement of TXNIP expression level was performed in the placenta of patients with GDM in this study. To mimic TXNIP expression degree in GDM placenta of the real world as much as possible, doxycycline (Dox) was introduced as a molecular switcher to induce the expression of TXNIP. TXNIP is upregulated via the addition of Dox in TetTXNIP cells. On the other side, TXNIP was knocked out via CRISPR-Cas9. The motility of placental trophoblast cells and its effects on the structure as well as the function change of the GDM placenta cell model was observed. Furthermore, TXNIP was found to regulate Vimentin by inducing transcription factor STAT3 and its phosphorylation.

## Materials and methods

### Clinical samples

The study has been approved by the Ethics Committee of Inner Mongolia People’s Hospital (No. 202,203,407 L). The subjects of patients with GDM and the health control were enrolled strictly. The enrolled criteria for GDM patients are detailed as follow: firstly, pregnant women aging from 25 to 40 years; secondly, and GDM was diagnosed based on the plasma glucose value of oral glucose tolerance test (OGTT-75 g) during 24–28 weeks of gestation, and the cut-off level is: fasting plasma glucose ≥ 5.1 mmol/L, and/or 1 h and/or 2 h after glucose loading ≥ 10 mmol/L and  ≥ 8.5 mmol/L, respectively [13]; lastly, the cesarean section was adopted. The enrolled criteria of the health control subjects were consistent with those of the GDM group except the GDM relating items. On the other hand, to avoid the influences caused by other clinical characteristics other than GDM pathological status, the exclusion criteria are detailed as follow: firstly, patients with chronic obstructive pulmonary disease, heart disease, hypertension, liver and kidney disease, chronic hepatitis B and other chronic diseases; secondly, obesity, type 2 diabetes mellitus or accompanied by obvious mental abnormalities; thirdly, multiple pregnancies, and other complications of pregnancy. All the subjects have signed the research-informed consent. All the details of the subjects were listed in Table 1.

**Table 1:** Clinical characteristics of the subjects

Clinical characteristics	GDM (n = 16) M SD	NOR (n = 32) M SD
N (Total = 48)	16	32
Age at delivery (years)	33.83 5.0	31.84 4.13
Pregestational BMI (kg/m2)	28.92 4.63	29.24 4.13
Pregestational overweight (BMI ≥ 25 kg/m2)	83.33%	84.37%
0 min blood glucose during OGTT (mmol/liter)	5.32 0.78	4.53**** 0.24
60 min blood glucose during OGTT (mmol/liter)	9.74 1.99	7.16**** 1.36
120 min blood glucose during OGTT (mmol/liter)	7.9 1.69	6.68** 0.96
HbA1C (mmol/liter)	5.98 0.72	NA
Placenta grading	II	II
Insulin treatment (IT)	14	NA
Calorie-restricted (CR)	2	NA

M: Mean, SD: standard deviation, **p < 0.01, ****p < 0.0001; NA: Normal

The placental samples were obtained from the GDM patients and the health control immediately after delivery. For each patient, pieces of the placental villous tissue were taken at the location of 2 cm away from the root of umbilical cord, washed twice with PBS, and stored respectively. Part of samples were snap-frozen in liquid nitrogen before stored at -80° Celsius degrees, the rest of the samples were fixed at 4% paraformaldehyde, used for immunohistochemistry.

### Immunohistochemistry

The frozen samples were sectioned to perform the immunofluorescence. The procedure was the same as the one in the previous study [12]. The primary antibodies are as follows: rabbit monoclonal anti-TXNIP (dilution 1:100, CST, cat#14,715), rabbit monoclonal anti-Vimentin (dilution 1:100, CST, cat#5741) and rabbit monoclonal anti-Cytokeration 7 (dilution 1:1000, Abcam, cat#ab68459) [14].

### Construction of the stable TXNIP overexpression cell lines

Dox inducible overexpressed TXNIP HTR-8/SVneo cells (TetTXNIP) were established as well as the control group using the tetracycline-regulated transgene expression (Tet-On) gene regulation system. The pTet-On and pTRE2pur-TXNIP were purchased from Clontech, China (No. 631013). HTR-8/SVneo cells were trypsinized and seeded in 6-well plates (Corning, Acton, MA) at a density of 2.5 × 10^5^ cells/well. Before transfection, cells were cultured in RPMI 1640 medium for 24 h. 1 µg of pTet-On containing a regulatory protein gene (rtTA) and a selection gene (anti G418) was transfected into HTR-8/SVneo cells by Lipofectamine LTX and Plus Reagent (Invitrogen, 2135022) according to the manufacturer’ s directions. Transfected cells were screened with 1 µg/µl of G418 (Gibco, Thailand, 2,121,291) for 30 d and isolated as monoclonal using flow cytometry (Beckman Coulter, MoFlo Astrios EQs, United States). 21 G418 resistant clones called Tet-On cell lines were isolated and freezed for the second transfection. Then, 1 µg of pTRE2pur-TXNIP containing integrated tetracycline-responsive promoter TXNIP cDNA and anti puromycin gene was transfected into the Tet-On cell lines with the same method. Transfected cells were screened with 15 µg/ml of puromycin (EMD Millipore Corp, Billerica, MA, United States) for 10 d and isolated as monoclonal using flow cytometry. The TXNIP expression levels were detected by RT-qPCR and western blot assay in those puromycin resistant clones in response to 10 µg/ml of Dox ( Sigma, United States, SLCC329) for 24 h.

### Construction of the stable knockout cell lines

Two single-guide RNAs (sgRNAs) targeting exon 1–8 of the TXNIP were designed by https://zlab.bio/guide-design-resources website. The pX330 vector (Addgene, 42,230) was used to produce pX330-TXNIP-gRNA1 plasmids and pX330-TXNIP-gRNA2 plasmids (Table 2). HTR-8 cells were placed in a six-well plate, and when the cells had grown to 70% fusion. Four plasmids (0.25 µg per plasmid), Lipofectamine LTX (12 µL) and Plus Reagent (2.4 µL) (Invitrogen, 2,135,022) were transfected in HTR-8 cells. The single cells were identified using flow cytometry (Beckman Coulter, MoFlo Astrios EQs, United States) at 24 h after transfection. Collected cells were plated into 96-well plates and monitored for the growth of single colonies. After 1 week, the cultured monoclonal cells were transferred from the 96-well plate to the 24-well plate for further culture until the 24-well plate was full. The DNA were extracted and PCR products were determined via Sanger sequencing. Cell lines with the 377 bp DNA fragment cut out were selected. The TXNIP expression levels were verified by RT-qPCR and western blot assay.

**Table 2:** Sequence of sg RNAoligo

Name	Sequence (5’- 3’)
sgRNA1 forward	CACCGCCTGAAAAGGTGTACGGCAG
sgRNA1 reverse	AAACCTGCCGTACACCTTTTCAGGC
sgRNA2 forward	CACCGCTTTGAAGTAGTGGATCTGG
sgRNA2 reverse	AAACCCAGATCCACTACTTCAAAGC

### Quantitative RT-PCR

Total RNA was extracted from cells and tissues using Trizol Reagent (Invitrogen, 15,596,018), and reverse transcription was performed using PrimeScript™ RT reagent Kit with gDNA Eraser (TaKaRa, RR047A). The CFX96™ Real-Time System (Bio Rad, UK) was used for the amplification reaction using the recommended reaction conditions. The gene primer sequences are described in Table 3. β-actin was used for normalization, and 2–ΔΔCt was used to calculate the relative expression.

**Table 3:** Primer sequences of the detected genes

Name	Sequence (5’- 3’)
TXNIP forward	TGCCACCACCGACTTATACTGA
TXNIP reverse	CCTGCTGACCACCTCCTACA
E-cadherin forward	GAATGACAACAAGCCCGAAT
E-cadherin reverse	GACCTCCATCACAGAGGTTCC
N-cadherin forward	GGTGGAGGAGAAGAAGACCAG
N-cadherin reverse	GCATCAGGCTCCACAGT
Vimentin forward	GTCCACCCGCACCTACAG
Vimentin reverse	CGAGAAGTCCACCGAGTCCT
β-actin forward	TCCCTGGAGAAGAGCTACGAGC
β-actin reverse	TGCCACAGGACTCCATGCCCAG

### Western blotting analysis

Total protein was extracted using RIPA buffer (Solarbio, #R0020), containing protease inhibitor phenylmethylsulfonyl fluoride (PMSF) (Solarbio, #P0100). The concentration of the extracted protein was determined by a Pierce™ BCA Protein Assay Kit (Thermo Scientific, 23,227). Western blotting analysis was carried out according to the experimental method as reported. Specific antibody–protein complexes images were captured via a gel imaging system (GE Healthcare Life Scientific, Amersham Imager 600) and quantified by Analysis Software Version1.0 (GE Healthcare Life sciences). The primary antibodies are as follows: anti-TXNIP (dilution 1:1000, Abcam, ab215366), anti-E-cadherin (dilution 1:1000, CST, #3195s), anti-N-cadherin (dilution 1:1000, CST, #13116s), anti-Vimentin (dilution 1:1000, Abcam, ab8978), anti-Stat3 (dilution 1:1000, CST, #9193), anti-Phospho-Stat3 (dilution 1:1000, CST, #9134), and anti-β-actin (dilution 1:1000, Santa, sc-32,233).

### Cell proliferation assay

Cell proliferation was measured via the CellTiter 96 Non-Radioactive Cell Proliferation Assay Kit (Promega, G4001). Approximately 2000 HTR-8 cells were inoculated into 96-well plates, and 100 µL culture medium was added to each well. When the cell confluence reached 80%, the cells were treated with 15 µL Dye Solution to each well and then the plates were incubated for 1–4 h in a humidified CO^2^ incubator. 100 µL Solubilization Solution was added to each well and the absorbance at 570 nm was recorded using a microplate reader.

### Cell migration and invasion assay

Cell migration and invasion was detected by a scratch-wound assay using IncuCyte. Cells were plated into 96-well plates at a density of 4 × 104 cells/well. Cells were transfected with pCMV3-TXNIP or pCMV3-NCV after 12 h. The 96-well wound maker was used to generate a wound in a monolayer of confluent cells. Matrigel matrix (Corning, 35,624) should be added into the 96-well plates in the invasion experiment. Migrated and invasion data and images of cells were automatically acquired by the IncuCyte Imaging System (Incucyte S3, Sartorius, United States) at 4 h intervals.

### Apoptosis assay

Cell apoptosis was determined with FITC Annexin V Apoptosis Detection Kit I (BD Biosciences, 556,547). Cells were washed three times with cold PBS and resuspended in 1 X binding buffer, followed by incubation with FITC Annexin V and PI for 15 min at room temperature in the dark. Apoptotic cells were quantified by flow cytometry (Beckman Coulter, Navios, United States).

### Statistical analysis

Statistical analysis was performed using Graphpad Prism 6.0 (Graph-Pad, USA), and the results were presented as mean ± SEM. Significant differences were determined by *t*-test, if *p* < 0.05 indicated that the difference was statistically significant.

## Results

### TXNIP was widely expressed in GDM placenta

**Fig. 1:**
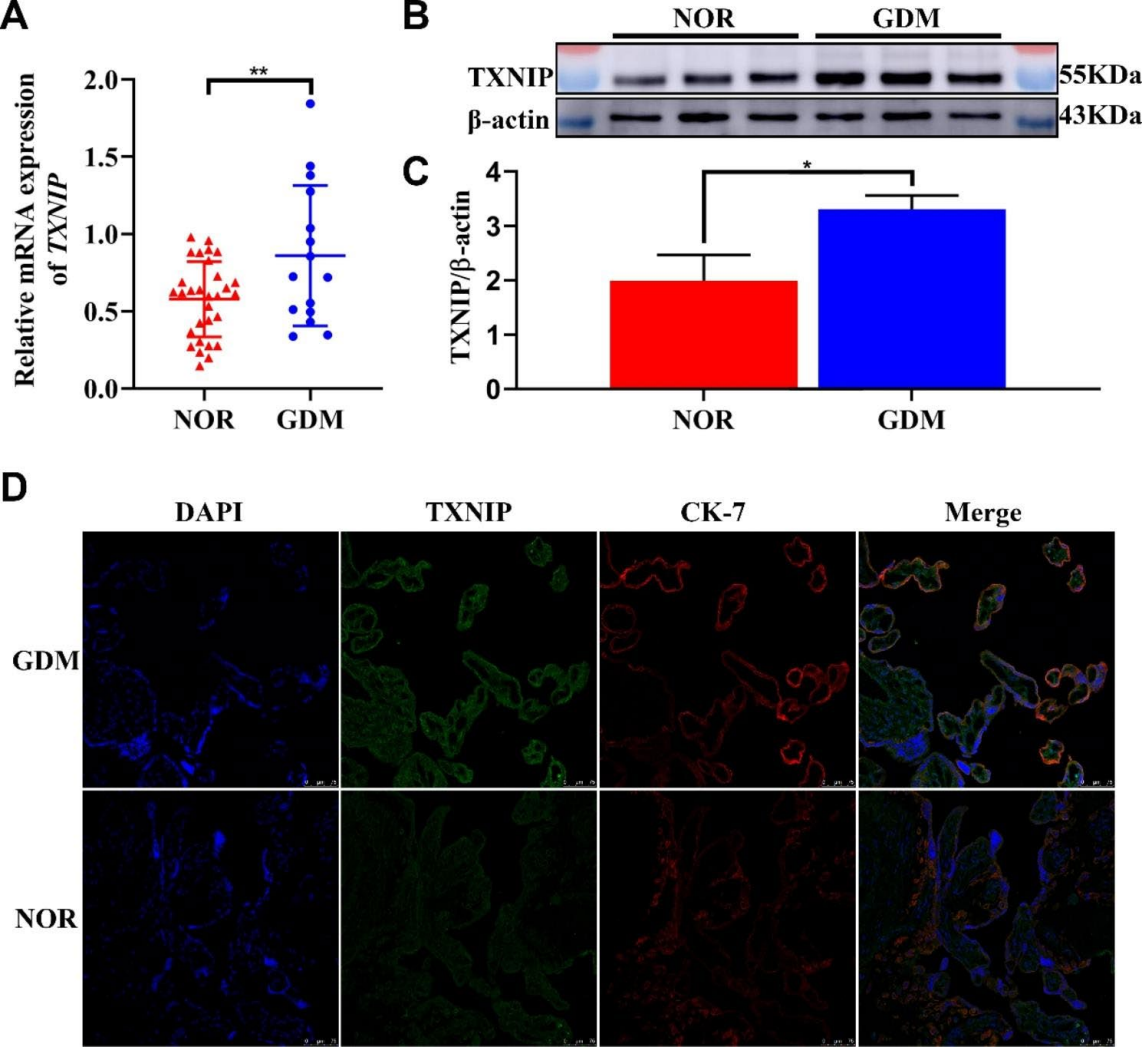
Widely Expression and the Localization of TXNIP in GDM Placenta **A**: The mRNA expression of TXNIP in placenta from healthy and GDM group by RT-qCR, respectively; **B-C**: The protein expression of TXNIP in placenta from healthy and GDM by western blot, respectively; **D**: Immunoflurescence analysis of TXNIP protein expression and the localization in the placenta from healthy pregnancy and GDM patients. Bar = 75 μm. *, compared with the NOR, and *p < 0.05. **p < 0.01. Results were expressed as mean ± SEM

The difference of TXNIP protein expression and its localization in the placenta between the healthy pregnant group and the GDM group was analyzed via RT-qPCR (Fig. 1A), western blot (Fig. 1B-C), and immunofluorescence (Fig. 1D). As shown in Fig. 1C, TXNIP protein localized at the cytotrophoblast cells, which were defined by the positive expression of cytokeratin 7 (CK-7). The expression of TXNIP protein and mRNA level significantly increased in the GDM group compared with those in the normal pregnancy group (Fig. 1A, B and C). Among patients with GDM, there was no statistical difference in TXNIP expression between insulin-treated and caloric restriction only (Supplementary Fig. 1); Furthermore, we found that TXNIP mainly expressed at cytotrophoblast cells shown by the immunofluorescence assay.

### Maternal and placental characteristics in the study population

There was no statistically difference in gestational age, BMI, pregestational overweight, placenta grading between the patients with the GDM and the control group. Whereas, the plasma glucose level of OGTT in GDM group is statistically significant higher than that in the control group at all time points. The glucose level of the GDM at three time points was 5.32 (0.78), 9.74 (1.99), and 7.9 (1.69), respectively; the glucose level of the control group at three time points was 4.53 (0.24), 7.16 (1.36) and 6.68 (0.96), respectively (Table 1).

### Overexpression of TXNIP significantly enhanced the invasion and migration capabilities of HTR-8/svneo cells

**Fig. 2:**
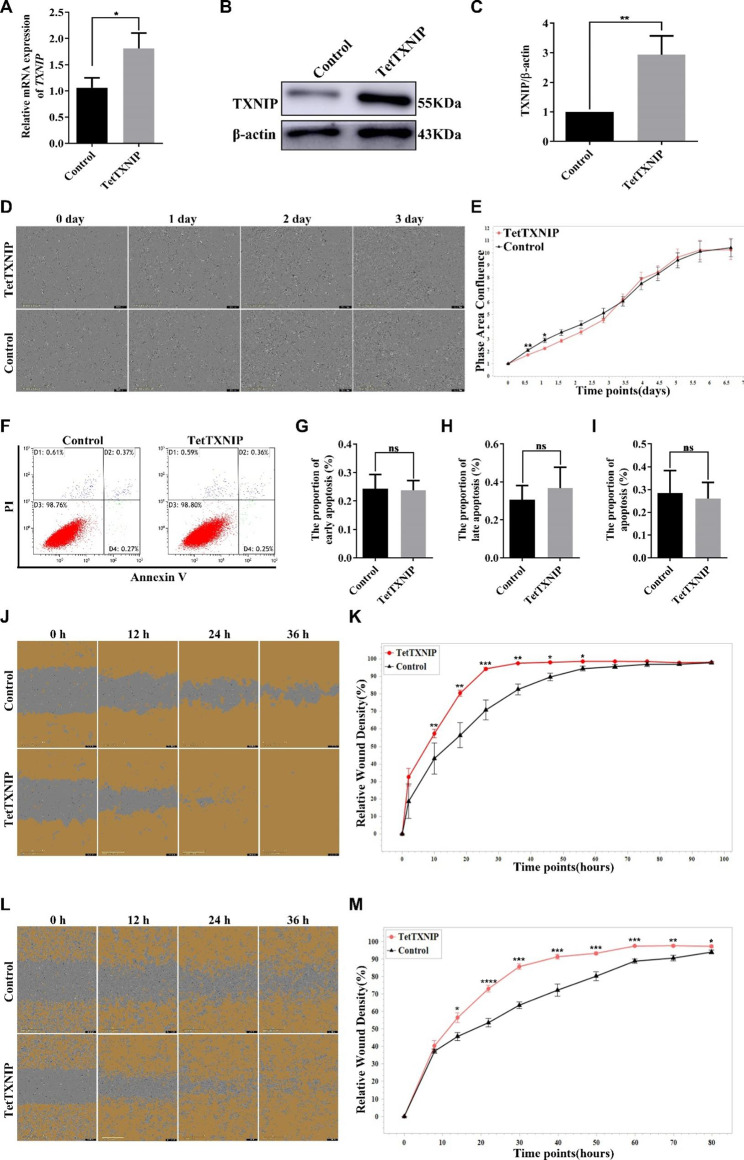
Overexpression of TXNIP Significantly Enhanced the Invasion and Migration Capabilities of HTR-8/svneo cells **A**: The mRNA expression of TXNIP in TetTXNIP-edited HTR-8/SVneo cells induced by Doxvia RT-qPCR. **B-C**: The expression of the TXNIP protein was analyzed by western blot in TetTXNIP-edited HTR-8/SVneo cells induced by Dox. **D-E**: The proliferative ability of TetTXNIP cells induced by Dox was monitored by IncuCyte. **F-I**: Cell apoptosis of TetTXNIP cells induced by Dox was analysed via flow cytometry. **G**: The early apoptosis. **H**: The late apoptosis. **I**: The total apoptosis. **J-K**: The cell migratory ability was elevated in the TetTXNIP cells induced by Dox via the scratch wound assay. **L-M**: The cell invasion ability was elevated in the TetTXNIP cells induced by Dox via the scratch wound assay. *, compared with the control, and *p < 0.05, **p < 0.01, ***p < 0.001, ****p < 0.0001. Results were expressed as mean ± SEM

To mimic the placental situation in the patients with GDM, Doxycycline-inducible overexpressed TXNIP HTR-8/SVneo cells (TetTXNIP) were established; the tetracycline (Tet)-on system was used in the control group. To induce TXNIP overexpression, TetTXNIP cells were treated with 10 µg/ml doxycycline (Dox). The mRNA expression of TXNIP in TetTXNIP cells was 1.7 times higher than that in the control cells (Fig. 2A). In terms of TXNIP protein expression, the expression of TXNIP protein in TetTXNIP cells was 2.9 times higher than that in the control cells (Fig. 2B-C). The proliferation of TetTXNIP cells were observed and recorded via Long-term dynamic cell observation and functional analysis system (IncuCyte). The number of TetTXNIP cells was monitored from 0 to 120 h and counted with an integrated metric of IncuCyte. Compared to the control group, there was no significant statistical difference observed on the proliferation ability (Fig. 2D-E). After proliferation assay, we further investigated the effects of TXNIP on HTR-8/Svneo cell apoptosis using flow cytometry analysis. It turned out that there was no significant difference between the two groups in the proportion of cells both in early apoptosis and late apoptosis (Fig. 2F-I). The migratory ability of TXNIP in HTR-8/SVneo cell line was detected by scratch test, and IncuCyte was used for recording and analysis. As shown in Fig. 2J, the wound was basically healed at 36 h in the TetTXNIP cells, while the control cells were not healed obviously. Moreover, TetTXNIP cells facilitated cell migration began to be evident statistically at 12 h, and significant acceleration in wound closure was observed in TetTXNIP cells at 26 h. The trend continued till migration cell density achieved 100%, indicating that the migratory ability increased significantly in the TetTXNIP cells, As shown in Fig. 2K (*p* < 0.05). The scratch width of the TetTXNIP group and the control groups was approximately equal at 0 h, and the wounds of the TetTXNIP group were healed at about 36 h, while those of the control group were not healed (Fig. 2L). The invasive capability of TetTXNIP cells increased dramatically compared with the control cells from 10 h till the end of the observation, As shown in Fig. 2M (*p* < 0.05).

### Knockout of TXNIP suppresses the invasion and migration capabilities of HTR-8/svneo

**Fig. 3:**
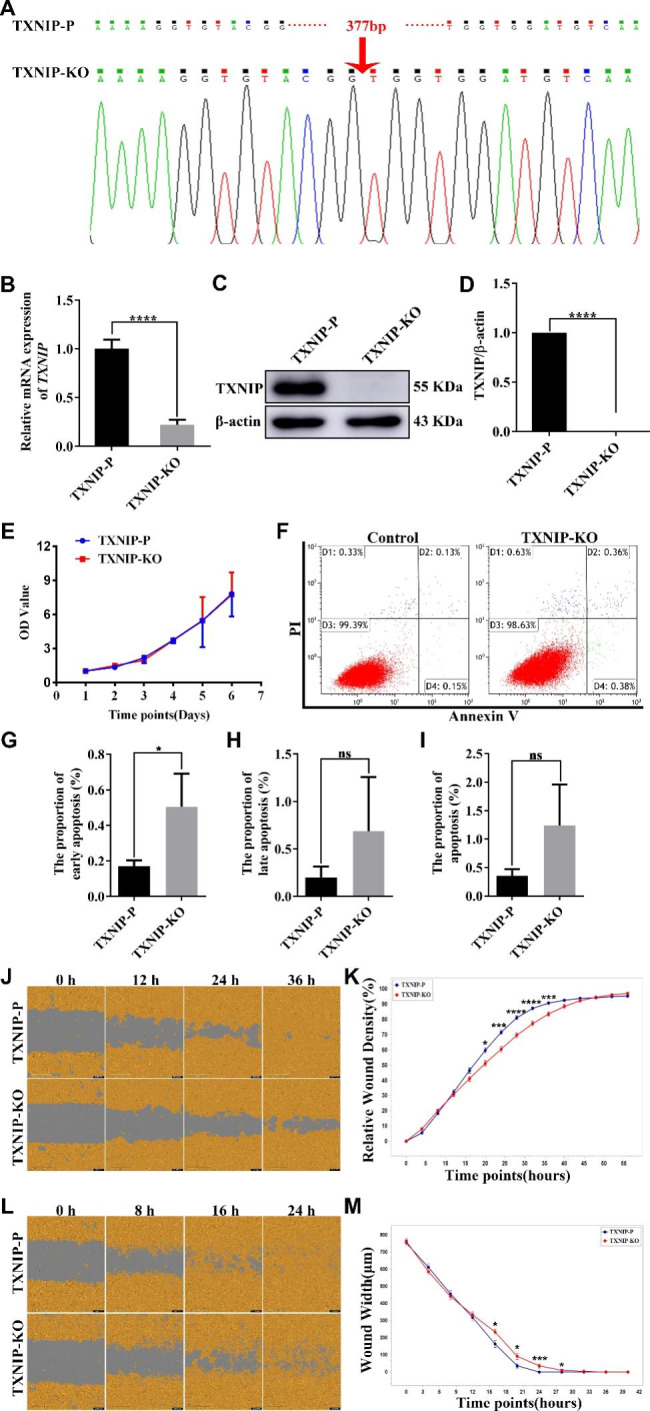
TXNIP Knockout Inhibited the Migration and Invasion of HTR-8/Svneo Cells, but had no Effect on Proliferation and Apoptosis **A**: TXNIP was knocked out in a biallelic manner in HTR-8/Svneo cell lines, Sanger sequencing results of TXNIP in TXNIP-P and TXNIP-KO HTR-8/Svneo cell lines. 377 bases of TXNIP were deleted in TXNIP-KO cell lines. **B**: RT-qPCR identified TXNIP mRNA expression changes in TXNIP-KO cell line. **C-D**: Western blots identified TXNIP protein expression changes in TXNIP-KO cell line. **E**: The proliferative ability of HTR-8/SVneo cells was determined by MTT assay. **F-I**: Flow cytometry analyze of cell apoptosis progress. **G**: The early apoptosis. **H**: The late apoptosis. **I**: The total apoptosis. **J-K**: The cell migratory ability was attenuated in the TXNIP-KO by the scratch wound assay. **L-M**: The cell invasion ability was attenuated in the TXNIP-KO group by the scratch wound assay. *, comparison of TXNIP-KO cells with TXNIP-P cells; and *p < 0.05, ***P < 0.001, ****P < 0.0001. Results were expressed as mean ± SEM

To furhter confirm the vital role of TXNIP on cell invasion and migration in trophoblast cells, TXNIP gene in parent HTR-8/Svneo cells was knocked out using CRISPER-Cas9-sgRNA gene editing system. Sanger sequencing results showed that 377 base pairs (bp) in TXNIP knockout cell lines were deleted successfully (Fig. 3A). Subsequently, RT-qPCR and western blot were used to detect the knockout efficiency. As shown in Fig. 3B, the mRNA expression of TXNIP in TXNIP-KO cells was 4.5 times lower than that in the parental cells (TXNIP-P). The TXNIP protein in TXNIP-KO cells was almost undetectable (Fig. 3 C-D). Knocking out of TXNIP did not affect HTR-8/Svneo cells proliferation (Fig. 3E) and apoptosis (Fig. 3F) compared with the control group. The apoptosis including early (Fig. 3G), late (Fig. 3H) and toal apoptosis (Fig. 3I) represented in thegraphs. However, the wound in the TXNIP-P cells basically healed at 36 h, while the migratory ability decreased significantly in the TXNIP-KO cells (Fig. 3J). Quantitative analysis of relative wound density (Fig. 3K) showed that knockout of TXNIP significantly attenuated the migratory ability of HTR-8/SVneo cell line (*p* < 0.05). A significant decrease in the invasion rate with TXNIP-KO was shown in Fig. 3L. At 0 h, the scratch widths of TXNIP-P group and TXNIP-KO group was approximately equal. At 24 h, the TXNIP-P group had healed, while the TXNIP-KO group did not until about 36 h (*p* < 0.05) (Fig. 3M). These results were compatible with observations in TetTXNIP cell lines; thus, the significance of TXNIP in trophoblast cells is firmly affirmed.

### TXNIP involved in the cellular morphology of HTR-8/svneo

**Fig. 4:**
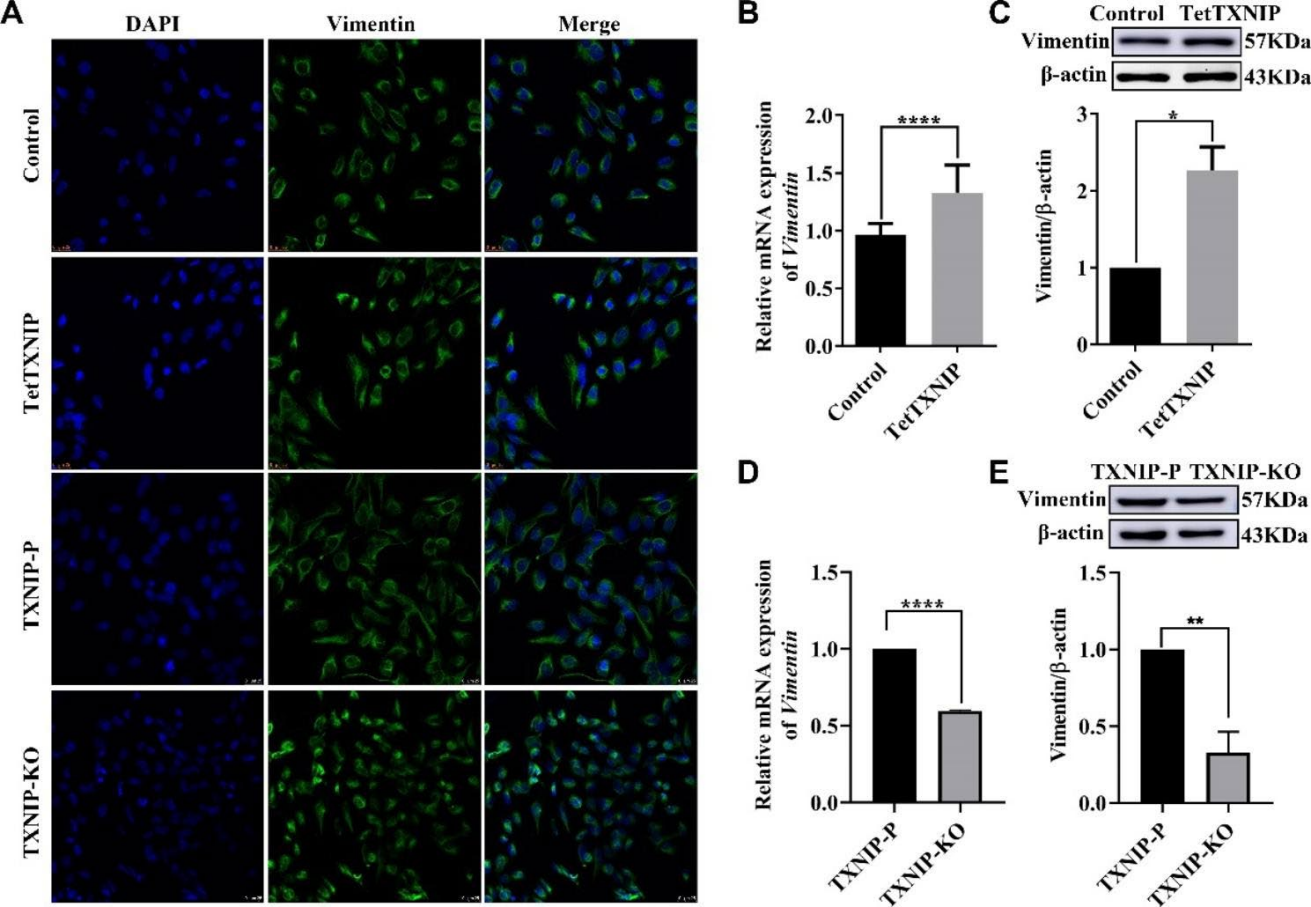
TXNIP Involved in the cellular morphology of HTR-8/Svneo **A**: Immunofluorescence analysis of Vimentin protein expression in the TetTXNIP, Control, TXNIP-P and TXNIP-KO cell lines. Bar = 25 μm. **B**: The mRNA expression of Vimentin in TetTXNIP cells induced by Dox via RT-qPCR. **C**: The expression of the Vimentin protein was analyzed by western blot in TetTXNIP cells induced by Dox. **D**: RT-qPCR identified Vimentin mRNA expression changes in TXNIP-KO cells. **E**: Western blots identified Vimentin protein expression changes in TXNIP-KO cells. *, compared with the Control or the TXNIP-P cells, and * *p* < 0.05, **p < 0.01, ****p < 0.0001. Results were expressed as mean ± SEM

The following experiments were carried out in these two pairs of groups, which is TetTXNIP and Control, TXNIP-P and TXNIP-KO, respectively. The cell morphology was compared via immunofluorescence staining separately. The TetTXNIP cells displayed a densification morphology than that in the control. The morphology of TXNIP-KO cells was shrinked, and showed a disintegration cytoplasm compared with the cytoplasm of TXNIP-P (Fig. 4A). Vimentin expression level was detected by RT-qPCR (Fig. 4B, D) and western blot analysis (Fig. 4C, E). The mRNA and protein expression level of Vimentin was significantly decreased in TXNIP-KO compared with those in the TXNIP-P (*p* < 0.0001), and increased in TetTXNIP compared with the control (*p* < 0.01). These results indicated that TXNIP expression level is associated with morphology.

### TXNIP affects the migration and invasion of HTR-8/SVneo cell line by regulating the expression of EMT-related factors

**Fig. 5:**
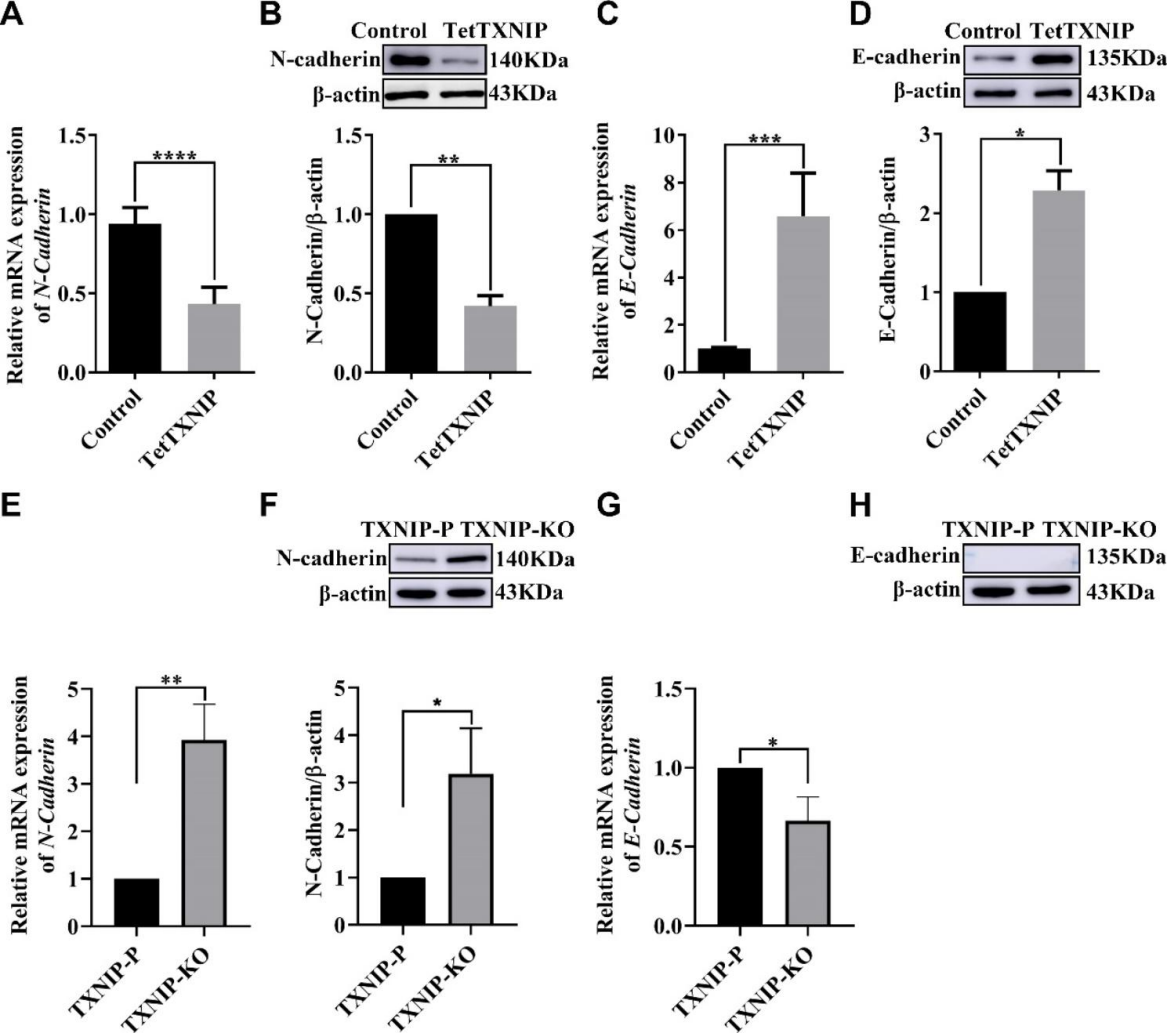
Effects of TXNIP on EMT-Related Genes in HTR-8/Svneo Cell Lines **A**: The mRNA expression of N-Cadherin in the TetTXNIP cells. **B**: The protein expression of N-Cadherin in the TetTXNIP cells. **C**: The mRNA of E-Cadherin in the TetTXNIP cells. **D**: The protein expression of E-Cadherin in the TetTXNIP cells. **E**, **F**: The mRNA expression of N-Cadherin and its protein expression in the TXNIP-KO cells. **G**, **H**: The mRNA expression of E-Cadherin and its protein expression in the TXNIP-KO cells. *, compared with the control or the TXNIP-P cells, and *p < 0.05, **p < 0.01, ***p < 0.001, ****p < 0.0001. Results were expressed as mean ± SEM

We assumed that TXNIP affects the migration and invasion of HTR-8/SVneo cell line by regulating the expression of EMT-related factors. As expected, the mRNA and protein expression of the E-cadherin increased in TetTXNIP cells compared with those in the control group (Fig. 5C-D). However, N-cadherin was decreased in TetTXNIP cells (Fig. 5A-B). On the contrary, both the mRNA and protein expression level of N-cadherin increased in the TXNIP-KO cells compared with the TXNIP-P cells (Fig. 5E-F). The mRNA expression level of E-cadherin was reduced in TXNIP-KO cells compared with those in the TXNIP-P cells, but there was no band detected in both TXNIP-KO cells and TXNIP-P cells (Fig. 5G, H). These results indicated that the expression of such EMT-related genes as vimentin, N-cadherin and E-cadherin is the important driving force of enhanced infiltration ability in placental trophoblast cells.

### STAT3 is regulated by TXNIP through phosphorylation alteration in HTR-8/Svneo cells

**Fig. 6:**
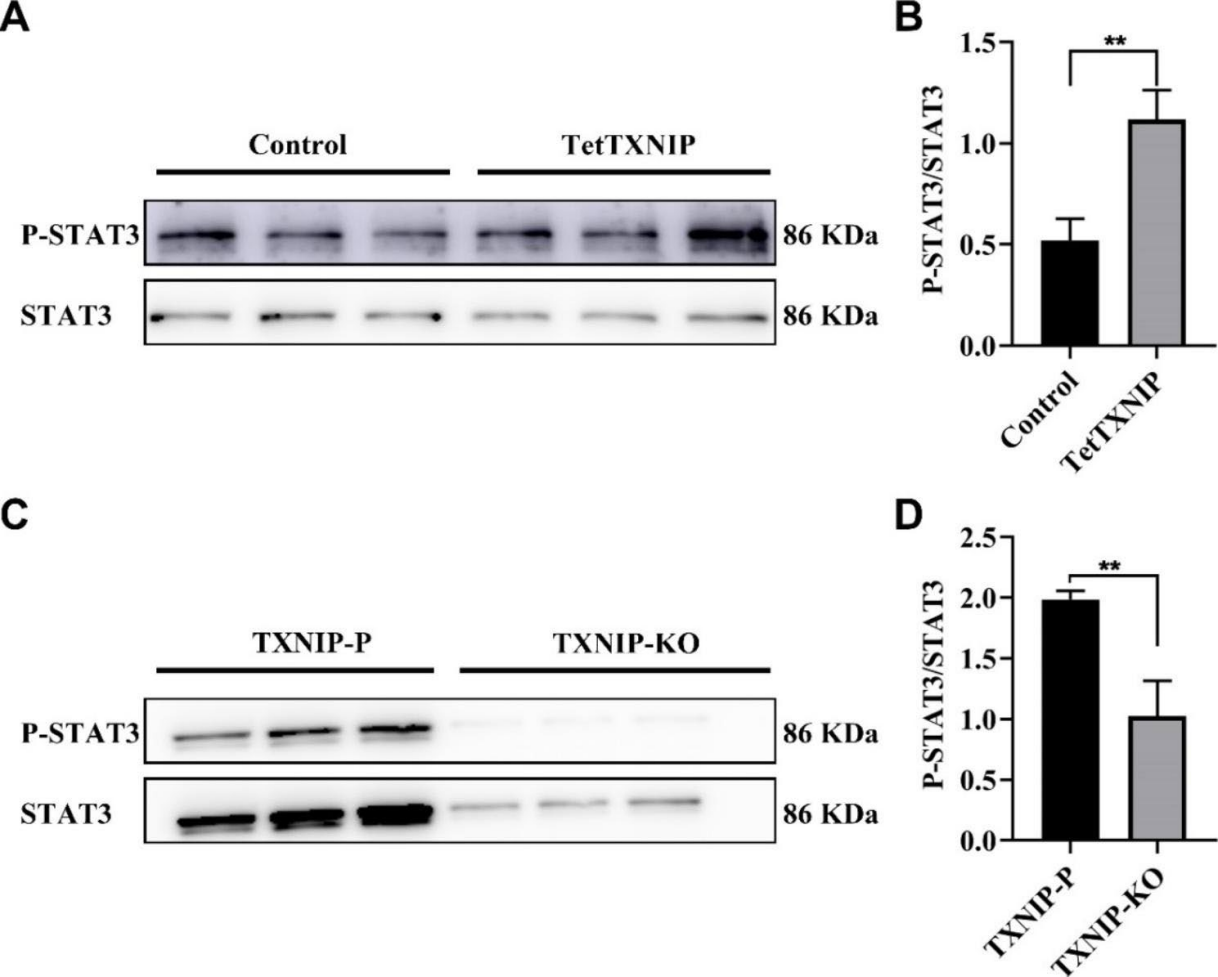
TXNIP Induced Cell Invasion was Dependent on the STAT3 in HTR-8/Svneo Cell Lines **A-B**: The p-STAT3/STAT3 protein levels were determined by western blot in TetTXNIP cells. **C-D**: The p-STAT3/STAT3 protein levels were determined by western blot and statistical analyses in TXNIP-KO cells. *, compared with the control or the TXNIP-P cells, **p < 0.01. Results were expressed as mean ± SEM

We further determined the expression of STAT3 and its phosphorylation (p-STAT3) using western blot in TetTXNIP and TXNIP-KO cells. It showed that the expression level of p-STAT3 was significantly increased in the TetTXNIP cells compared with the Control group (Fig. 6 A), relative gray scale of western blot analysis results in (Fig. 6B). Meanwhile, there was no statistically change in STAT3 expression level between the TetTXNIP cells and the Control group (Supplementary Fig. 2). By contrast, knocking out of TXNIP resulted in the decreased p-STAT3 in TXNIP-KO cells (Fig. 6 C), relative gray scale of western blot analysis results in (Fig. 6D).

## Discussion

Placenta is the temporary organ during the pregnancy. It could provide the oxygen and abundance nutrition to the fetus and bring the metabolic waste to the maternal circulation [14, 15]. It is the proliferation and migration of the trophoblast cells which could provide the biological structure bases for the normal development of the placenta [14]. Therefore, the biological function of placenta is the essential element to sustain the normal pregnancy process. GDM is a common complication during pregnancy [16]. Besides hyperglycemia, more researchers have found that placenta of the GDM patients was impaired, but the most of the underlining mechanisms are waiting to be explored [17].

In this study, we verified that the expression of TXNIP, both the mRNA level and the protein level, in the placenta of the GDM patients was higher than those in the healthy control. TXNIP locates in the trophoblasts of the placenta. In the previous study, the expression of TXNIP was observed via immunofluorescence rather than the quantitative measures [12]. Thus, the detection of TXNIP was more accurate in the present study, which was showing that instead of 10 times or more, the expression of TXNIP tripled in GDM placenta in the real world. The interesting phenomenon is when TXNIP was transiently overexpressed up to 10 fold (in the previous study), cell proliferation and migration were slowed down and apoptosis increased; when the high expression of TXNIP was controlled by the Tet-on system up to 2–3 times, the cell proliferation was basically unchanged, and the migration and invasion accelerated. When TXNIP was knocked out via CRISPR-Cas9, general apoptosis and late apoptosis were unchanged statistically compared with that in the TXNIP-P cells, but early apoptosis was increased. It is reported that TXNIP is well-known regulator of intracellular ROS, and ROS soared in TXNIP-KO mice [18], In our experiments, either 10 fold over-expressed TXNIP in the previous study, or knocked-out TXNIP in the present study, stress has been loaded on the cellular activity, various stress factors such as ROS increased, inspiring apoptosis. In our experiments, the statistical difference presented in the early apoptosis instead of the late or general apoptosis in TXNIP-KO cells, showing that the early apoptosis cells were major population of the apoptosis cells, the reason why there was no statistical difference in the late or general apoptosis might lies in the detection time point. We chose the second day of culture after the cells were recovered. In the following research, dynamic consistent observation will be performed to figure out the curve of different phase of apoptosis. In the present study, this trend only could prove that knocked-out TXNIP drive the apoptosis.

TXNIP is multifunctional in regulating glucose and lipid metabolism, even influence the proliferation of vascular smooth muscle cells [19–22]. In our research, TXNIP expression is also found increased in GDM placenta; however, the upstream gene is not studied. Yi et al. confirmed that TXNIP was increased in placental samples of GDM patients, which was consistent with ours. It is reported that MiR-17-5p could up regulate the expression of TXNIP, improved glucose uptake of HTR-8/Svneo cells by TXNIP/NLRP3 axis [23].

The phenotype change of the TXNIP-edited cells were quite obvious. The cell size became larger when TXNIP was over expressed, companied with the accelerated migration and invasion; while smaller when it was knocked out, with slower migration and invasion. Such phenomenon enlightened us to think of Vimentin, a member of cytoskeletal protein [24]. As expected, the expression of Vimentin was positively correlated with the expression of TXNIP, which could explain the underlying mechanism of the reason why the cell size, the migration and the invasion would change. Also, this mechanism has been reported in most of cancers [24–26]. The expression level of Vimentin is positively correlated with cell invasion, especially in epithelial cancers and preeclampsia placenta [27, 28]. Whereas, Vimentin is the essential protein of cellular intermediate filaments, its expression change will affect the cell shape, the ability of cell migration, cell adhesion and cell signaling activities accordingly. In tissue culture scratch wound experiments, knocked-out Vimentin in fibroblasts showed slower migration when compared with their corresponding wild-type [29, 30].

Many studies show that the dysregulation of STAT3 could cause the pathological processes of placenta during placental development [31]. As a cytoplasmic transcription factor, STAT3 regulates many key genes involved in cell functions such as apoptosis, differentiation, proliferation and migration through dephosphorylation activation. Relative Studies show that STAT3 activity is related to the placenta trophoblast cell invasion [31]. In preeclampsia placenta tissues, the change of STAT3 expression is related to the altered migration and the invasion of the trophoblast [32, 33]. In this study, we clarified that STAT3, as a key downstream regulator of TXNIP, affects the migration and invasion of placental trophoblast cells. Many investigations have indicated that STAT3 is constitutively activated in many cancers and played a main role in tumor growth and metastasis [34, 35]. In studies of liver cancer metastasis, STAT3 was founded to be cooperated with Snail-Smad3/TGF-B1 signaling pathway, promoting cell migration [36]. Our study showed that the phosphorylated STAT3 increased in the TetTXNIP cells; while decreased in the TXNIP-KO cells. Studies [37] demonstrated that STAT3 was phosphorylated more in GDM placenta samples than the one in the health control, and the TXN-NFkB-STAT3 pathway was verified to be blamed for. That is, TXNIP and STAT3 play an important role in the infiltration of human placental trophoblast cells into the uterine wall in GDM from the viewpoints of both our study and the other study. He et al. verified that TXNIP is bound to STAT3 directly, which means TXNIP could activate the STAT3 pathway exacerbates renal tubular epithelial fibrosis [38].

Because of the changed cell migration, we further detected the expression of N-cadherin and E-cadherin. those expressions and obtained a different trend; The expression of N-cadherin was down and E-cadherin was up in TetTXNIP cells; the opposite results appeared in the TXNIP-KO cells. Surprisingly, the changing trend of N-cadherin was consistent with that of Vimentin. Many mechanism are involved in the cell migration, involving cell adhesion, cell morphological change, and mechanical tension and so on [39, 40]. The expression of Vimentin was regulated by the cell skeleton and cell motivation regulating pathway besides the EMT mode [41]. Therefore, the expression of Vimentin, N-cadherin and E-cadherin are not necessarily supposed to conform to the classical trend as reported [42]. As for the relationship between TXNIP and Vimentin in the GDM placenta, there is no other similar reports found except for ours. Exploring the cell skeleton pathways regulating Vimentin besides TXNIP is the work we are planning to do. Although the increased invasion is benign in the cell modal with high TXNIP expression which simulates the TXNIP status in GDM, the degree of the placenta into the uterine wall would be over deepened; as a results, the exchange ability between maternal and fetus would altered accordingly [43], the microenvironment might change unexpected. This might be the pathological base for the placenta dysfunction in GDM. What’s more, this point will be testified in the larger scale of the GDM placenta samples in the next research.

In brief, the present study suggested that the expression of TXNIP in the GDM placenta was higher than the healthy ones; the high expression of TXNIP can regulate the STAT3 signaling pathway, changing the cell morphology of trophoblast cells via changing Vimentin, thus regulating the migration and invasion of the trophoblast cells. The findings is thought to be the reasons of the structural and functional alterations in GDM placenta. The results of the study might provide the theoretical evidence for the targeted therapy to repair the placenta function in GDM treatment in the future. Also, enlighten further research on TXNIP regulating network and the epigenetics.

## Electronic supplementary material

Below is the link to the electronic supplementary material.

**Additional file 1: Supplementary Figure 1**: The difference between the mRNA expression of TXNIP in calorie-restriced (CR) group (n=2) and calorie-restriced plus insulin treatment (CR+IT) group (n=14) of GDM placenta was analyzed by RT-qPCR (Supplementary Figure 1). The results showed that there was no statistically significant difference in TXNIP mRNA expression between the two groups (p=0.26).

**Additional file 2: Supplementary Figure 2:** Compared with the vector control group, the total protein expression of STAT3 in TetTXNIP group was not change.

**Additional file 3:** Western blot

## Data Availability

All data generated and analysed in the process of the study are included in this article and its supplementary information files.
