# Supplementary material for: High TXNIP expression accelerates the migration and invasion of the GDM placenta trophoblast
Source: BMC Pregnancy Childbirth. 2023 Apr 10;23:235. doi: 10.1186/s12884-023-05524-6 (PMC10084645; doi:10.1186/s12884-023-05524-6)
Supplement: Supplementary file 1 — Additional file 1: Supplementary Figure 1: The difference between the mRNA expression of TXNIP in calorie-restriced (CR) group (n=2) and calorie-restriced plus insulin treatment (CR+IT) group (n=14) of GDM placenta was analyzed by RT-qPCR (Supplementary Figure 1). The results showed that there was no statistically significant difference in TXNIP mRNA expression between the two groups (p=0.26). [file 12884_2023_5524_MOESM1_ESM.docx]

**Supplementary Figure 1**

The difference between the mRNA expression of TXNIP in calorie-restriced (CR) group (n=2) and calorie-restriced plus insulin treatment (CR+IT) group (n=14) of GDM placenta was analyzed by RT-qPCR (Supplementary Figure 1). The results showed that there was no statistically significant difference in TXNIP mRNA expression between the two groups (*p*=0.26).

Supplementary figure 1. The mRNA expression of TXNIP in CR (n=2) and CR+IT groups (n=14) of GDM placenta. ns: no significant.
