# Supplementary material for: High TXNIP expression accelerates the migration and invasion of the GDM placenta trophoblast
Source: BMC Pregnancy Childbirth. 2023 Apr 10;23:235. doi: 10.1186/s12884-023-05524-6 (PMC10084645; doi:10.1186/s12884-023-05524-6)
Supplement: Supplementary file 2 — Additional file 2: Supplementary Figure 2: Compared with the vector control group, the total protein expression of STAT3 in TetTXNIP group was not change. [file 12884_2023_5524_MOESM2_ESM.docx]

**Supplementary Figure 2**

Compared with the vector control group, the total protein expression of STAT3 in TetTXNIP group was not change.

**
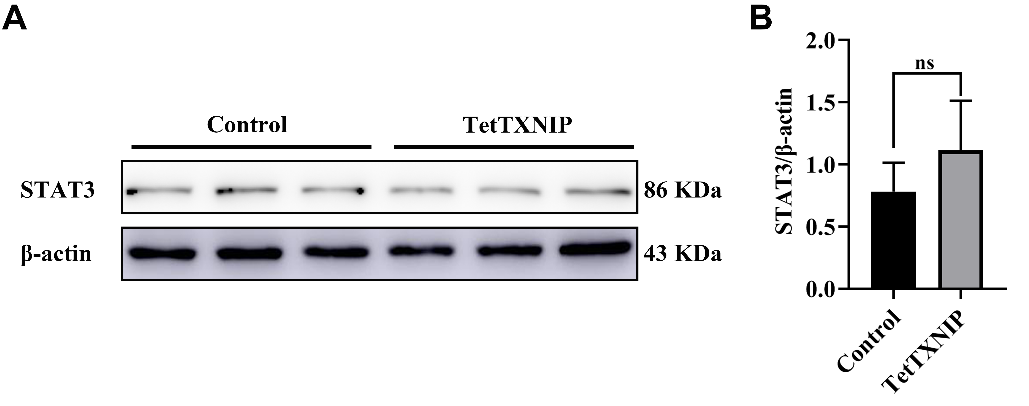
**

Supplementary figure 2. The expression of the STAT3 in TetTXNIP cells. Control: tetracycline (Tet)-on system HTR-8/SVneo cell line; TetTXNIP: overexpressed TXNIP HTR-8/SVneo cells. ns: no significant.
