# Supplementary material for: High TXNIP expression accelerates the migration and invasion of the GDM placenta trophoblast
Source: BMC Pregnancy Childbirth. 2023 Apr 10;23:235. doi: 10.1186/s12884-023-05524-6 (PMC10084645; doi:10.1186/s12884-023-05524-6)
Supplement: Supplementary file 3 — Additional file 3: Western blot [file 12884_2023_5524_MOESM3_ESM.docx]

**Western blot (Original) 1.**

**
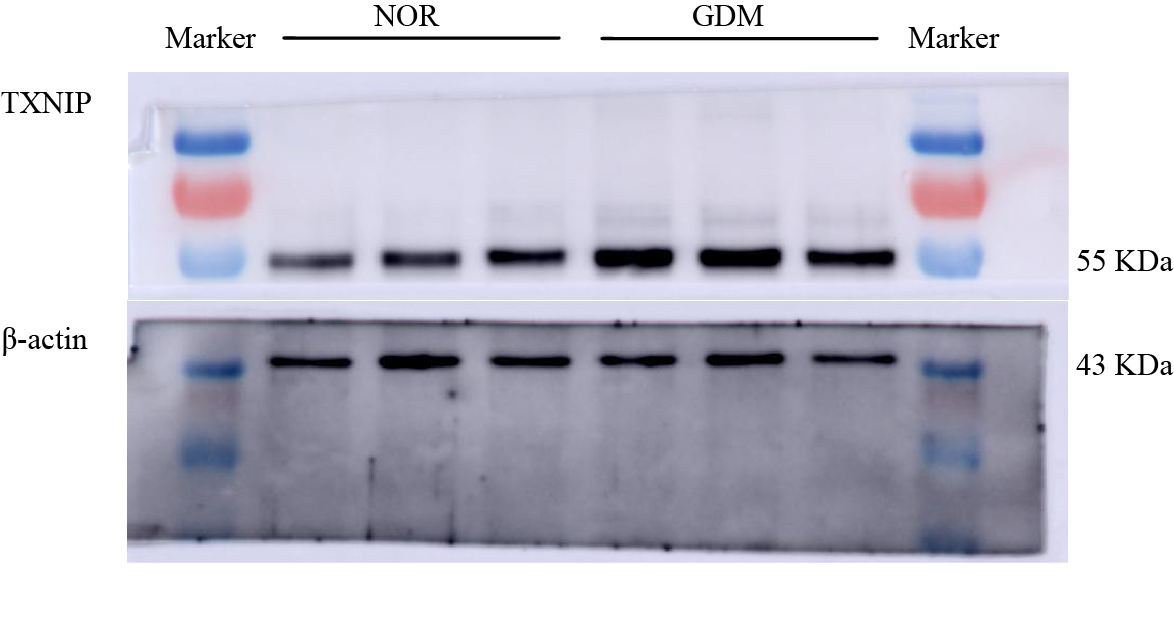
**

Western blot (Original) 1. The original scanning picture of Figure 1B. The picture illustrates the protein expression of the TXNIP in placentas from healthy pregnancy and GDM patients by Western blot. (NOR: healthy pergnancy placentas; GDM: GDM patients placentas)

**Western blot (Original) 2**


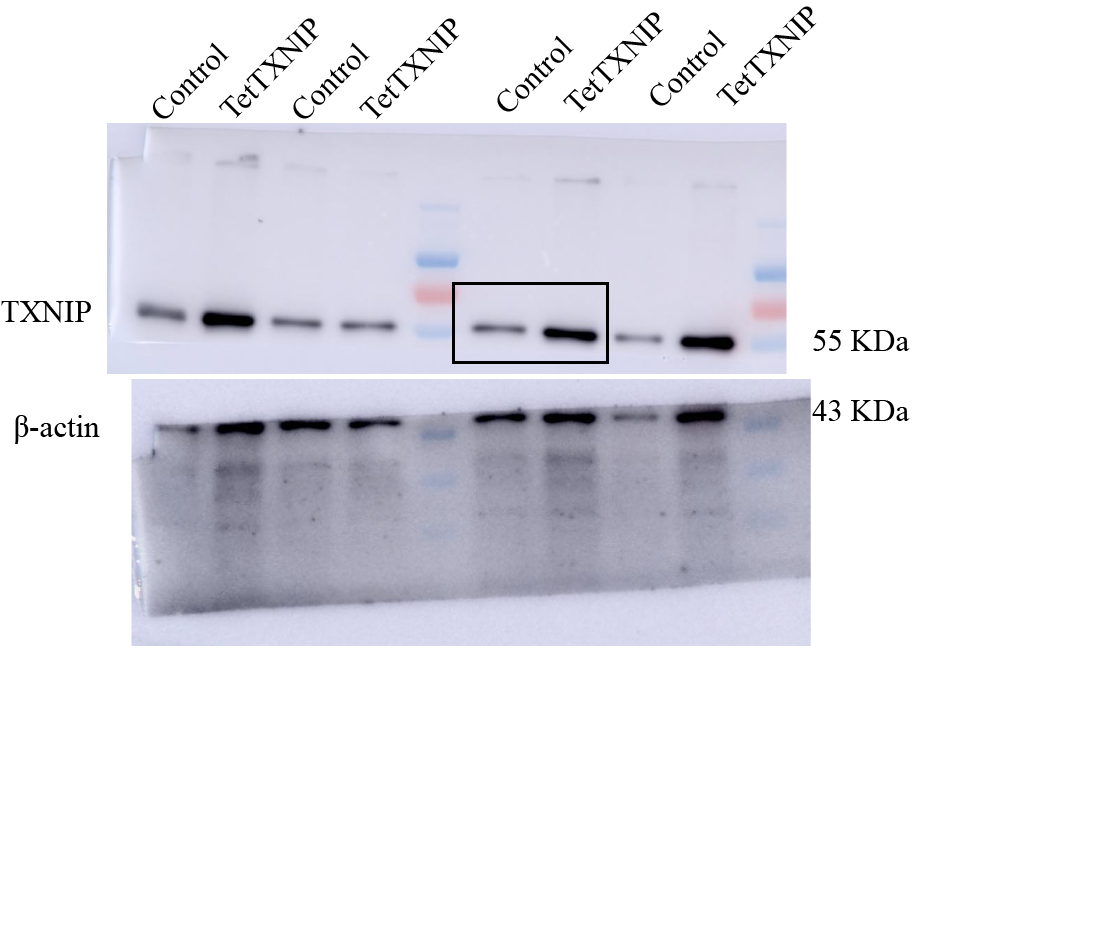


Western blot (Original)2. The original scanning picture of Figure 2B. The expression of the TXNIP protein was analyzed by Western blot in TetTXNIP-edited HTR-8/SVneo cells induced by doxycycline. (Control: tetracycline (Tet)-on system HTR-8/SVneo cell line; TetTXNIP: overexpressed TXNIP HTR-8/SVneo cells)

**Western blot (Original) 3.**


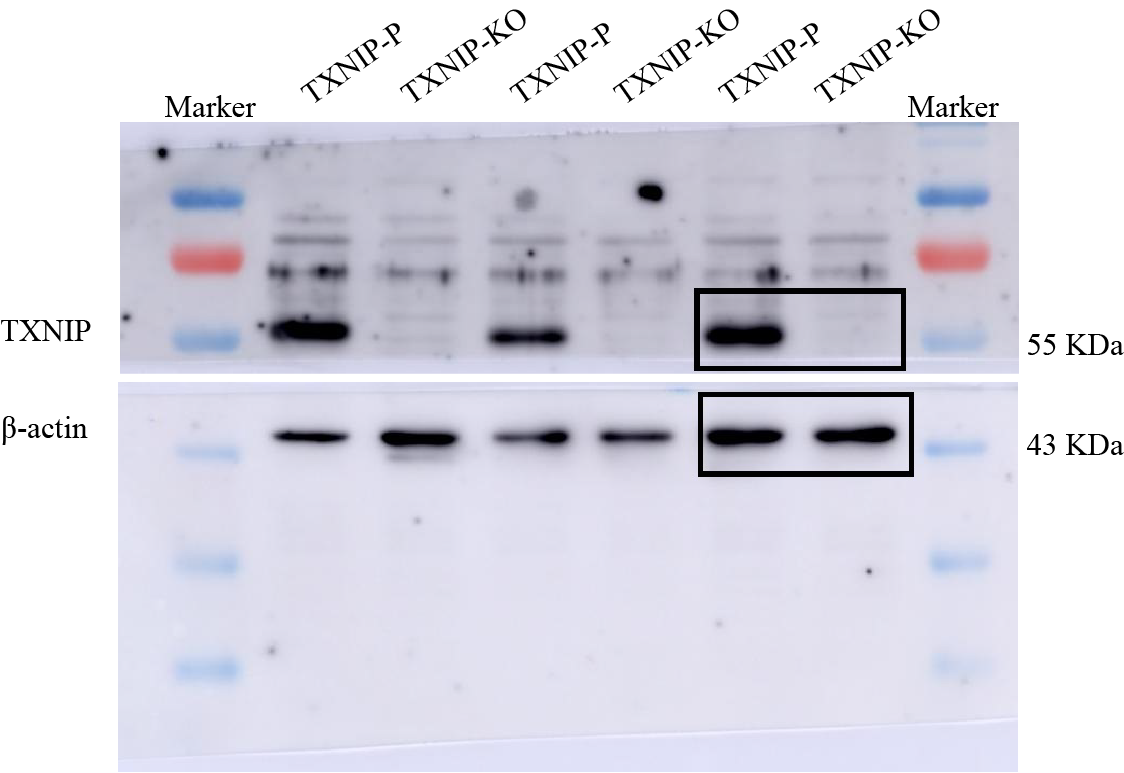


Western blot (Original)3. The original scanning picture of Figure 3C. Western blots identified TXNIP protein expression changes in HTR-8/SVneo cell line of TXNIP was knocked out. (TXNIP-P: HTR-8/SVneo cell line ; TXNIP-KO: HTR-8/SVneo cell line of TXNIP was knocked out)

**Western blot (Original) 4.**


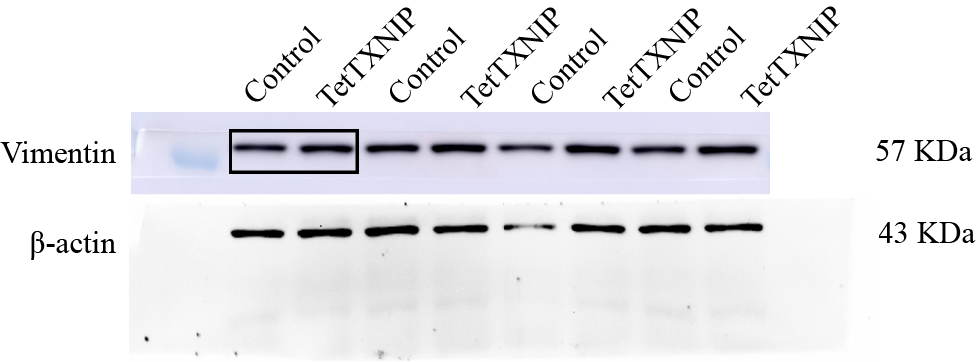


Western blot (Original) 4. The original scanning picture of Figure 4C. Western blots identified Vimentin protein expression changes in HTR-8/SVneo cell line of TXNIP was overexpression. (Control: tetracycline (Tet)-on system HTR-8/SVneo cell line; TetTXNIP: overexpressed TXNIP HTR-8/SVneo cells)

**Western blot (Original) 5.**

**
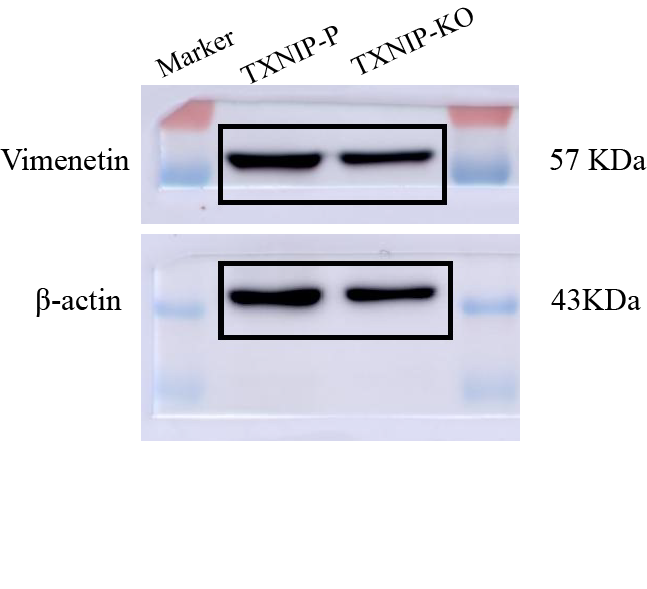
**

Western blot (Original) 5. The original scanning picture of Figure 4E. Western blots identified Vimentin protein expression changes in HTR-8/SVneo cell line of TXNIP was knocked out. (TXNIP-P: HTR-8/SVneo cell line ; TXNIP-KO: HTR-8/SVneo cell line of TXNIP was knocked out)

**Western blot (Original) 6.**


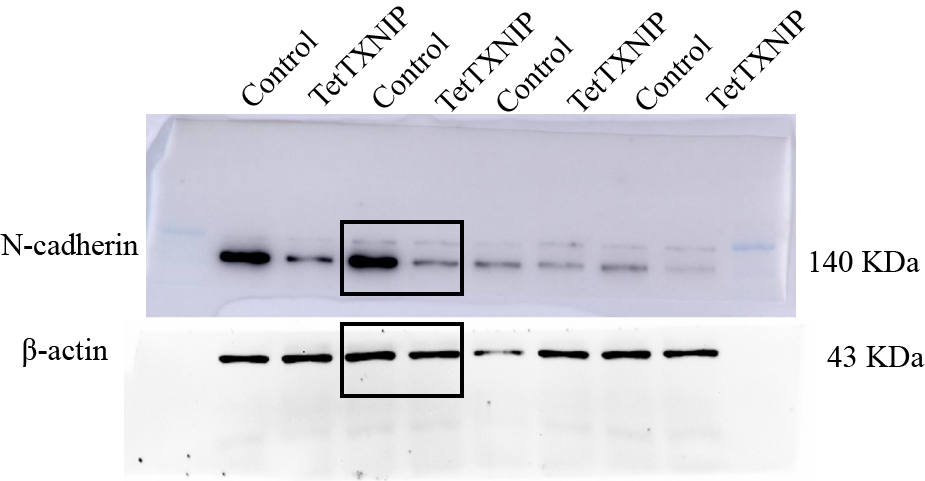


Western blot (Original) 6. The original scanning picture of Figure 5B. Western blots identified N-Cadherin protein expression changes in HTR-8/SVneo cell line of TXNIP was overexpression. (Control: tetracycline (Tet)-on system HTR-8/SVneo cell line; TetTXNIP: overexpressed TXNIP HTR-8/SVneo cells)

**Western blot (Original) 7.**

**
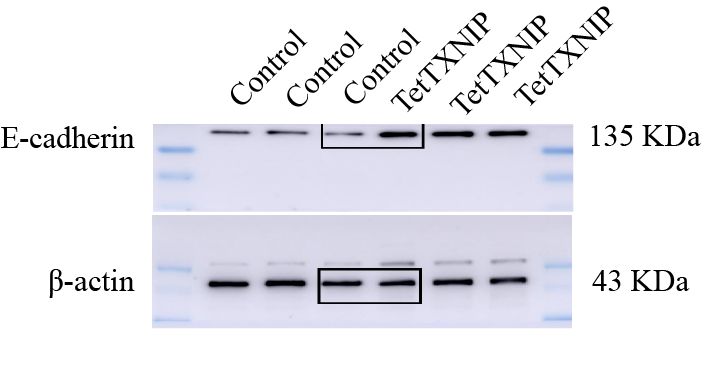
**

Western blot (Original) 7. The original scanning picture of Figure 5D. Western blots identified E-Cadherin protein expression changes in HTR-8/SVneo cell line of TXNIP was overexpression. (Control: tetracycline (Tet)-on system HTR-8/SVneo cell line; TetTXNIP: overexpressed TXNIP HTR-8/SVneo cells)

**Western blot (Original) 8.**

**
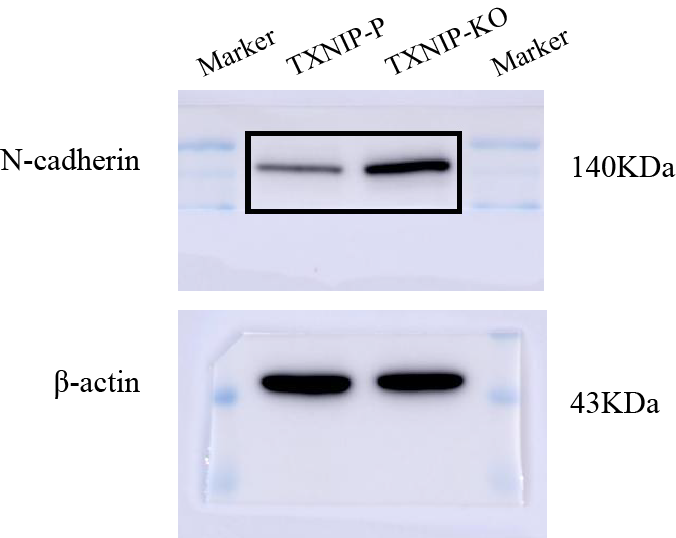
**

Western blot (Original) 8. The original scanning picture of Figure 5F. Western blots identified N-Cadherin protein expression changes in HTR-8/SVneo cell line of TXNIP was knocked out. (TXNIP-P: HTR-8/SVneo cell line ; TXNIP-KO: HTR-8/SVneo cell line of TXNIP was knocked out)

**Western blot (Original) 9.**

**
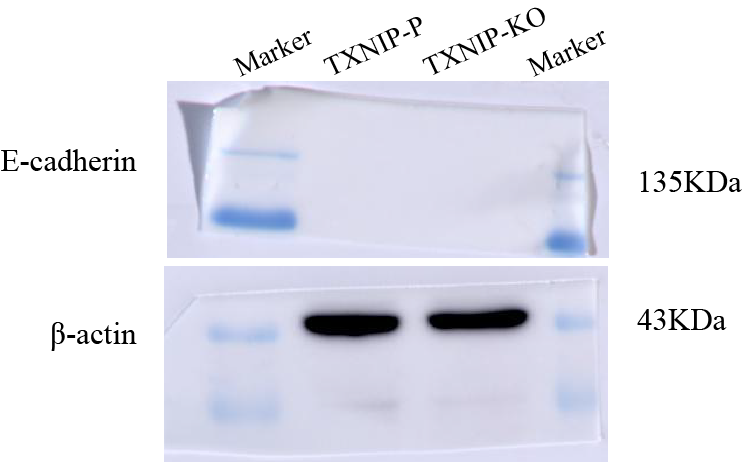
**

Western blot (Original) 9. The original scanning picture of Figure 5H. Western blots identified E-Cadherin protein expression changes in HTR-8/SVneo cell line of TXNIP was knocked out. (TXNIP-P: HTR-8/SVneo cell line ; TXNIP-KO: HTR-8/SVneo cell line of TXNIP was knocked out)

**Western blot (Original) 10.**

**
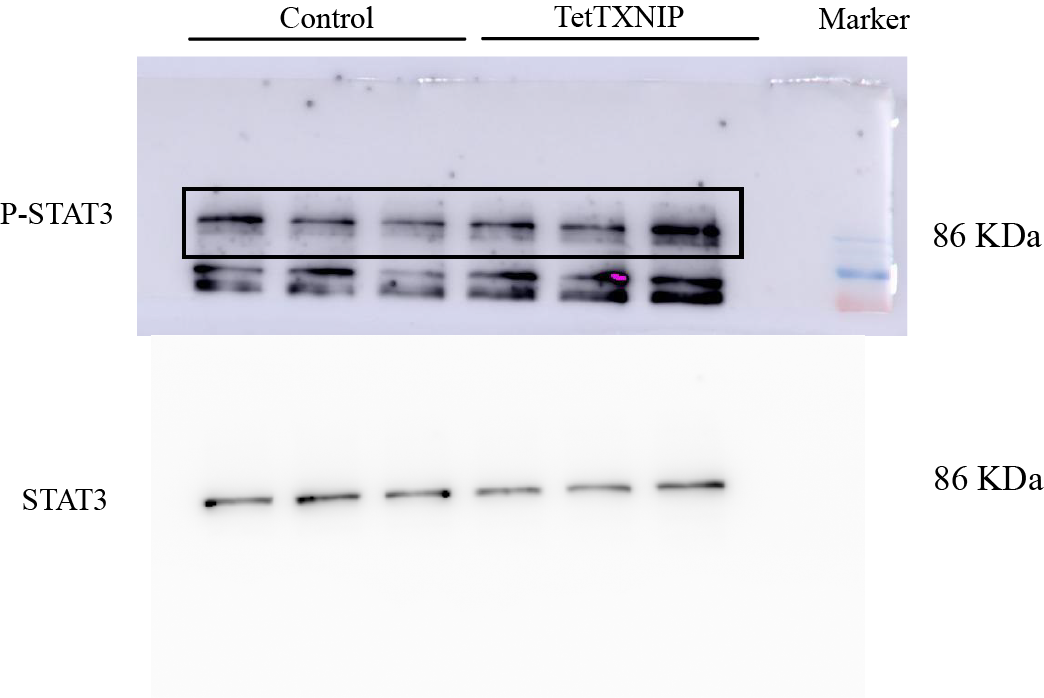
**

Western blot (Original) 10. The original scanning picture of Figure 6A. The expression of the P-STAT3/STAT3 protein was analyzed by Western blot and statistical analyses in TetTXNIP cells. (Control: tetracycline (Tet)-on system HTR-8/SVneo cell line; TetTXNIP: overexpressed TXNIP HTR-8/SVneo cells)

**Western blot (Original) 11.**

**
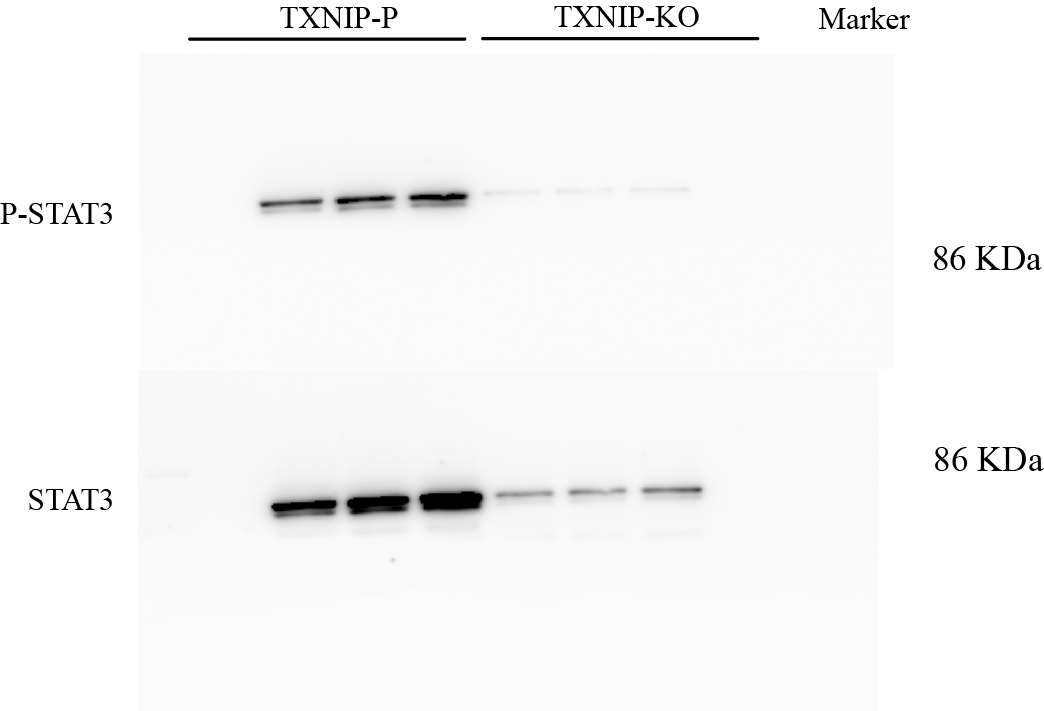
**

Western blot (Original) 11. The original scanning picture of Figure 6C. The expression of the P-STAT3/STAT3 protein was analyzed by Western blot and statistical analyses in TXNIP konckout cells. (TXNIP-P: HTR-8/SVneo cell line ; TXNIP-KO: HTR-8/SVneo cell line of TXNIP was knocked out)

**
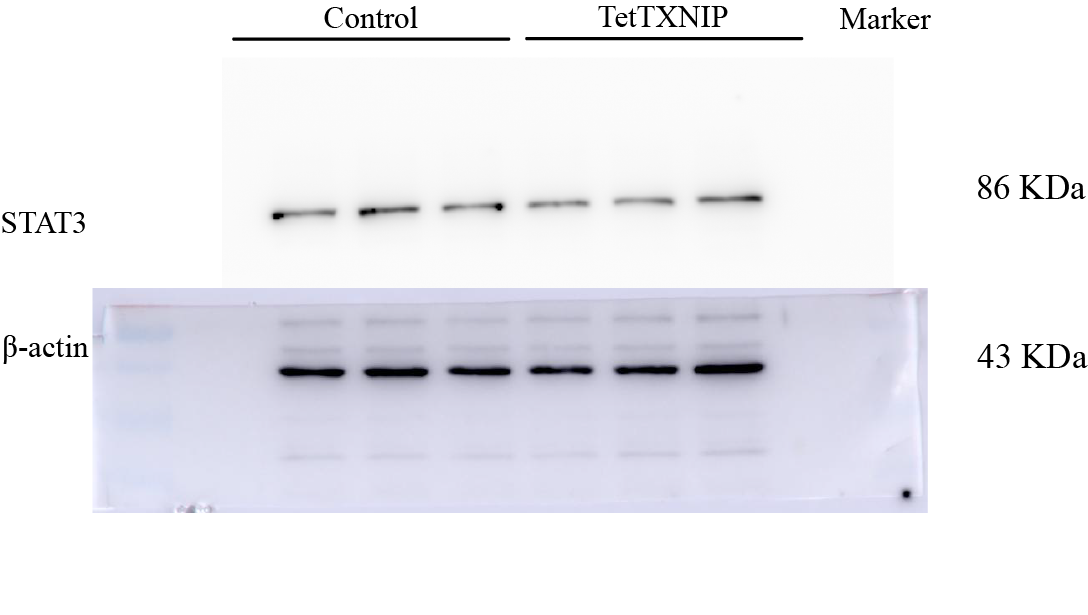
Western blot (Original) 12.**

Western blot (Original) 12. The original scanning picture of Supplementer Figure 2. The expression of the STAT3/β-actin protein was analyzed by Western blot and statistical analyses in TetTXNIP cells. (Control: tetracycline (Tet)-on system HTR-8/SVneo cell line; TetTXNIP: overexpressed TXNIP HTR-8/SVneo cells)
